# Separating cognitive and motor contributions to iADL difficulties in Parkinson’s disease

**DOI:** 10.3389/fnagi.2025.1732479

**Published:** 2026-01-08

**Authors:** Rekha Ravikumar, Marta Statucka, Melanie Cohn

**Affiliations:** 1 Krembil Brain Institute, Toronto Western Hospital UHN, Toronto, ON, Canada; 2 Department of Psychology, University of Toronto, Toronto, ON, Canada

**Keywords:** cognitive diagnosis, daily functioning, informant questionnaire, neuropsychological assessment, self-report questionnaire

## Abstract

**Background:**

Mild Cognitive Impairment (MCI) and dementia are distinguished by whether cognitive deficits interfere with independent performance of instrumental activities of daily living (iADLs). In Parkinson’s disease (PD) this distinction is challenging due to the combined impact of motor and cognitive symptoms on autonomy. To address this, we examine two methods aimed at isolating these contributions using the Functional Activities Questionnaire (FAQ): (1) a modified scoring method that classifies items as motor or cognitive to compute a ratio of the two contributors (FAQ_Q_), and (2) a novel, extended FAQ that captures patients’ and care-partners’ perspective to elucidate the cognitive burden experienced.

**Methods:**

We conducted a retrospective chart review of PD patients (*n* = 283) prior to Deep Brain Stimulation. We extracted ratings from the standardized and extended FAQ, cognitive diagnoses [PD-MCI: *n* = 164; cognitively normal (PD-CN) *n* = 119], neuropsychological test scores, and demographic and clinical variables. We examined the degree to which respondents attributed iADL difficulties to motor and cognitive symptoms, and whether these ratings matched the modified scoring method’s categorization. To validate this scoring method in our sample, we examined each standardized FAQ item’s relationship with measures of motor symptoms (UPDRS-III) and global cognition (DRS-II). Lastly, we derived a Reported Cognitive Burden (RCB) score from the extended FAQ and examined how it, and the FAQ_Q_, relate to cognitive status (PD-CN vs. PD-MCI) and performance on neuropsychological tests.

**Results:**

Patients and care-partners reported that iADLs were more limited by motor symptoms, even for items categorized as “cognitive.” Regression models did not achieve the same item classification as prior research using the modified scoring method. The RCB, but not the FAQ_Q_, was higher in PD-MCI than PD-CN and related to performance in attention and executive domains regardless of who provided the ratings.

**Conclusion:**

Patient’s and care-partner’s appraisal of the source of iADL difficulties was inconsistent with previous categorization of FAQ items in patients with more advanced PD. Our novel RCB offers a sensitive means of detecting mild functional changes related to cognition even in the presence of highly disabling motor symptoms, and may aid in establishing cognitive diagnoses in PD and other neurodegenerative disorders.

## 1 Introduction

Progressive cognitive impairment is a prominent non-motor symptom of Parkinson’s disease (PD) (Aarsland et al., 2021), and ranges from mild cognitive impairment (PD-MCI) (Litvan et al., 2012) to dementia (PDD) (Emre et al., 2007). Both diagnoses require evidence of difficulties on cognitive testing and reports of cognitive decline from the patient and/or care-partners. A PDD diagnosis additionally requires that cognitive impairments are sufficiently severe to interfere with the independent execution of instrumental activities of daily living (iADLs). Clinicians and researchers face several challenges in evaluating cognitively-mediated functional independence in PD, which can compromise diagnostic accuracy, and consequently, treatment planning and outcome measurements. First, the effect of cognitive difficulties must be distinguished from that of cardinal motor symptoms in PD (Cheon et al., 2015). Second, sampled activities should encompass a range of common iADLs to account for individual variability in roles and responsibilities (Kulisevsky et al., 2025). Third, severity of iADL difficulties should be captured as some changes in functioning may occur in PD-MCI, even prior to the onset of PDD (Kulisevsky et al., 2013).

Rating scales address some of these challenges by capturing the degree of difficulty across common iADLs. Although most iADL scales, which were primarily developed for use in Alzheimer’s disease, do not typically differentiate between the contributions of motor and cognitive symptoms to iADL impairments, some methods address this issue. One strategy has been to develop PD-specific iADL questionnaires, selecting activities with strong cognitive requirements (Brennan et al., 2016), and instructing responders to solely consider cognitive difficulties when rating functioning (Kulisevsky et al., 2013). Another approach has been to adapt existing iADL questionnaires either by eliminating items (Almeida et al., 2017) or modifying instructions (Cheon et al., 2015), although such modifications may alter the psychometric properties of these instruments. Alternatively, one group has modified scoring methods without changing test administration. Specifically, Becker et al. (2020) administered the standardized Functional Activities Questionnaire (FAQ) (Pfeffer et al., 1982), and categorized each item as motor, cognitive, or both. Their categories were based on each item’s degree of relationship with independent measures of motor symptoms [i.e., Unified Parkinson’s Disease Rating Scale (UPDRS-III] and global cognition [i.e., Montreal Cognitive Assessment (MoCA)]. The authors then derived motor and cognitive subscores by summing ratings for the respective item types and computed a quotient (FAQ_Q_ = cognitive subscore ÷ motor subscore). Patients classified as having cognitive iADL impairment (i.e., FAQ_Q_ > 1) showed greater impairments in attention/working memory and language, and were more likely to convert to PDD in the years that followed, highlighting the validity and possible prognostic value of this method (Becker et al., 2022a).

While innovative, this modified scoring approach relies on an indirect attribution of the underlying cause of the functional difficulty. Thus, it is unclear whether this corresponds to the respondent’s impression of the degree to which each type of symptom limits the patient’s ability to perform iADLs based on their lived experience. Second, given that the FAQ_Q_ was validated in patients in the early stages of PD, it is unclear whether the suggested threshold to identify the presence of cognitively-driven iADL impairment remains valid in patients with longer disease duration. Notably, the requirement that the cognitive subscore be greater than the motor subscore (FAQ_Q_ > 1) may not be met in patients with marked motor symptoms or fluctuations, such as those being considered for Deep Brain Stimulation (DBS). Accurate cognitive diagnosis is paramount in this group as PDD is considered a contraindication for DBS (Lang et al., 2006). Furthermore, although there is moderate agreement between patients’ and care-partners’ ratings on iADL questionnaires, including the FAQ (Becker et al., 2022b; Cholerton et al., 2020; Deck et al., 2019), the FAQ_Q_ was developed mainly from ratings by care-partners and did not expressly consider those of patients unless a care-partner’s ratings could not be obtained. It is common practice in PD to rely on care-partners’ reports, as patients’ insight may be compromised with increasing disease severity and cognitive impairment (Becker et al., 2022b; Deck et al., 2019). However, Cholerton et al. (2020) found that patient ratings are more sensitive than care-partner ratings in earlier stages of cognitive impairment (i.e., distinguishing normal cognition from PD-MCI). Lastly, several factors can influence care-partner ratings, including caregiver burden, quality or type of relationship to patient, education level, and cultural differences (Hackett et al., 2020), and knowledgeable care-partners may not always be available to complete rating forms. These findings highlight the importance of also examining patients’ self-report.

The present study examines both patients’ and care-partners’ evaluations of the source of iADL difficulties endorsed in pre-DBS patients with disabling motor symptoms and fluctuations. To this aim, the standardized FAQ was extended to include an explicit rating of the degree to which motor and cognitive symptoms contribute to iADL difficulties. This extension was administered after the standardized FAQ was completed to preserve the administration procedure and psychometric properties of the original form. We then explore whether ratings on the extended FAQ match the categorization used in the modified scoring method (Becker et al., 2020) in that items categorized as motor have higher reported limitation ascribed to motor symptoms than to cognitive symptoms, and conversely, items categorized as cognitive have higher reported limitation ascribed to cognitive symptoms than to motor symptoms. Thirdly, we examined the categorization of standardized FAQ items based on their degree of relatedness with independent measures of motor symptoms (UPDRS-III) and global cognition [Dementia Rating Scale-2 (DRS-2) (Jurica et al., 2004)]. This followed a similar approach to that used in previous research (Becker et al., 2020) in order to determine whether the same classification is achieved in PD patients with longer disease duration and more severe motor symptoms. Lastly, we derive a measure of cognitive iADL impairment based on patients’ and care-partners’ ratings on our extended FAQ. Specifically, we calculate the Reported Cognitive Burden (RCB) which measures the proportion of iADL difficulties ascribed to cognitive symptoms [i.e., cognitive ratings ÷ (cognitive + motor ratings)]. To validate our novel measure, we then examine the degree to which the RCB and the FAQ_Q_ relate to current cognitive status (cognitively normal vs. PD-MCI) and performance on neuropsychological tests across five cognitive domains.

## 2 Materials and methods

### 2.1 Participants

**FIGURE 1:**
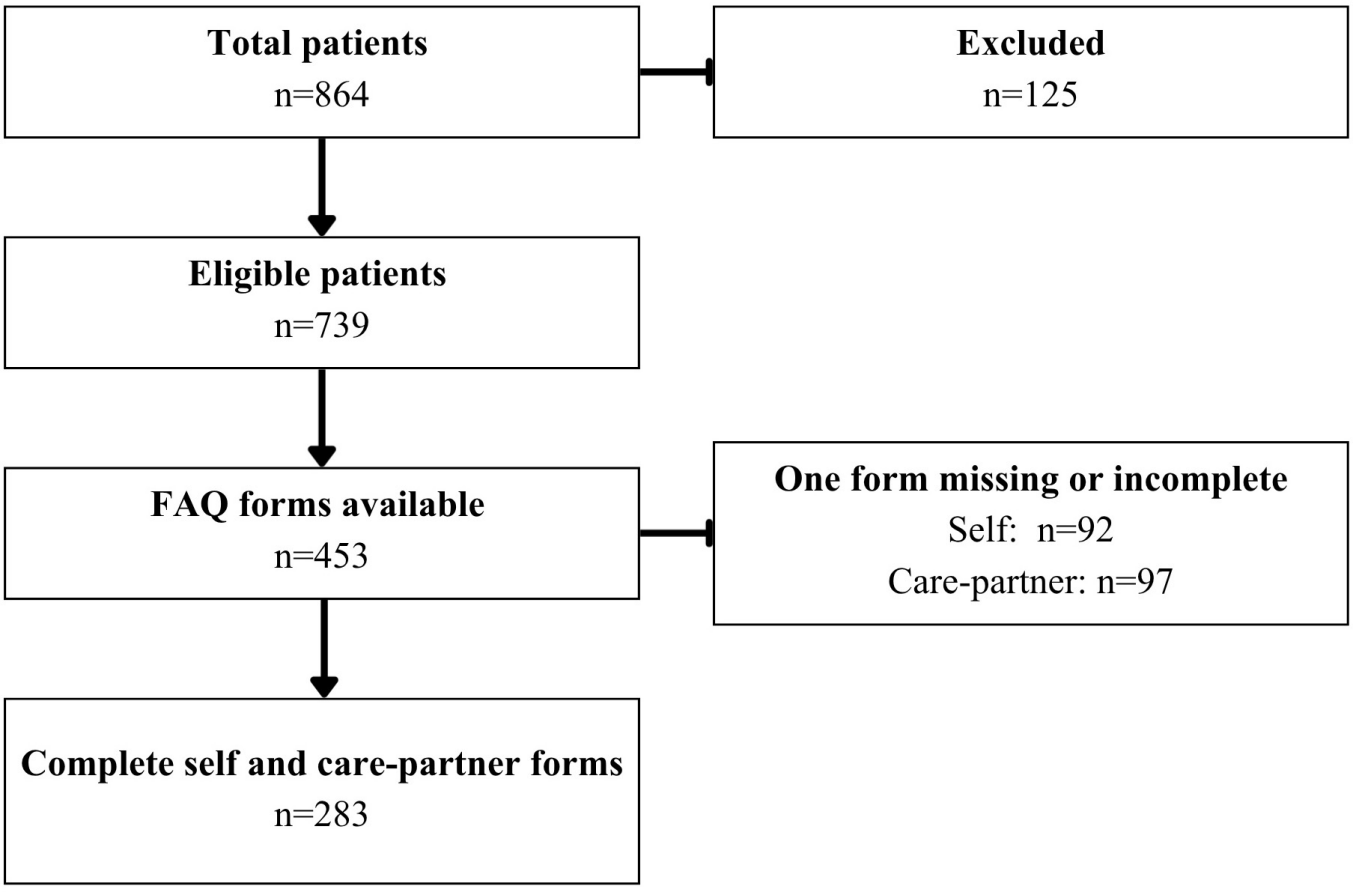
Flow diagram of patient inclusions and exclusions.

We conducted a retrospective chart review of PD patients who underwent comprehensive neuropsychological evaluation prior to consideration for DBS at Toronto Western Hospital between January 2009 and September 2020. The following exclusion criteria were applied: comorbid neurological disorders (e.g., stroke, seizures, moderate/severe traumatic brain injury, neurodevelopmental disorders), previous neurosurgery, severe psychiatric disorders (e.g., schizophrenia, personality disorders, substance abuse), PDD diagnosis based on level II MDS criteria (Emre et al., 2007), and missing or incomplete FAQ forms. After exclusions, 283 patients with both self- and care-partner-reported FAQ forms were included. A flow diagram outlining patient eligibility and exclusions is presented in Figure 1.

### 2.2 Demographic and clinical variables

Demographic variables including age, sex, and years of education, as well as PD duration (i.e., years since diagnosis) and levodopa equivalent daily dose (LEDD) were extracted to characterize our sample. To capture motor symptom severity, the Unified Parkinson’s Disease Rating Scale part 3 (UPDRS-III) (Fahn and Elton, 1987) was administered as part of standard clinical care while in the ON state within 12 months of the iADL assessment. In cases where the MDS-UPDRS-III (Goetz et al., 2008) was administered (*n* = 85), scores were transformed to match the original version (Hentz et al., 2015). Depression severity was determined using the Beck Depression Inventory II (BDI-II) (Beck et al., 1996) or the Geriatric Depression Scale (GDS) (Yesavage et al., 1982). Specifically, scores < 14 on the BDI-II and < 10 on the GDS were classified as no depression, mild levels corresponded to scores of 14–19 on the BDI-II or 10–19 on the GDS, and moderate-severe levels corresponded to scores of 20 or greater on both the BDI-II and GDS.

### 2.3 Neuropsychological assessment

Patients underwent a comprehensive neuropsychological evaluation from which data pertaining to iADL difficulties and cognitive functioning were extracted.

#### 2.3.1 iADL questionnaires

Patients and their care-partners completed the standard and extended versions of the FAQ to measure difficulties with iADLs. They first completed the standard FAQ which required them to rate the patient’s ability to perform 10 activities as normal or not applicable (0), has difficulty but does by themselves (1), requires assistance (2), or is dependent (3). Following this, they completed an extension of the questionnaire wherein patients and care-partners rated the degree to which each activity is limited by (a) motor symptoms (e.g., tremors, stiffness, freezing, walking and balance difficulties, poor handwriting, unpredictable OFF periods), and (b) cognitive symptoms (e.g., poor attention, impulsivity, poor judgment, changes in concentration, memory, or problem solving). Specifically, they indicated whether each symptom type was not at all limiting (0), mildly limiting (1), moderately limiting (2), or severely limiting (3). Since the aim was to examine the degree to which difficulties on the standardized FAQ were attributed to motor and cognitive symptoms, respectively, ratings of individual items on the extended FAQ were only included in analyses if the ability to perform each task on the standard FAQ received a score > 0 (i.e., excluding “normal” ability and “not applicable” responses).

Standard FAQ items were categorized according to scoring method proposed by Becker et al. (2020): Three items were labeled as cognitive [items 1 (Cheques and bills), 2 (Complex finances), and 9 (Remembering important events)] and five were classified as motor [items 3 (Shopping/errands), 4 (Hobbies/skills), 5 (Making coffee), 6 (Preparing meals), and 10 (Travel out of the house)]. We categorized items 7 (Tracking current events) and 8 (Attention to media) as “neutral” since they were included in both the motor and cognitive subscores used in the scoring method.

From the standardized FAQ, we calculated a total score from 0 to 30, and computed the FAQ_Q_ (Becker et al., 2020) as a measure of cognitive iADL impairment. From the extended FAQ, we calculated the average rating for motor and cognitive symptoms at three levels: (a) for all FAQ items grouped together, (b) for FAQ items grouped into the three categories (motor, cognitive, and neutral), and (c) for each FAQ item individually. Additionally, we derived a novel measure of cognitive iADL impairment using ratings on the extended FAQ that reflects the degree to which cognitive symptoms contribute to overall iADL impairment. If “Cog” represents the total score on the cognitive symptom section of the extended FAQ, and “Mot” represents the total score on the motor symptom section of the extended FAQ, then the Reported Cognitive Burden (RCB) for each patient is defined as:


$$R\,C\,B=\frac{C\,o\,g}{C\,o\,g+M\,o\,t}$$

#### 2.3.2 Neuropsychological assessment

##### 2.3.2.1 Global cognition

Global cognition was assessed using the DRS-2 (Jurica et al., 2004). Missing values were replaced by the group’s median (*n* = 4).

##### 2.3.2.2 Cognitive diagnosis

Patients were diagnosed with PD-MCI according to the MDS level II criteria (Litvan et al., 2012). Specifically, PD-MCI was diagnosed if they showed impairment on two or more neuropsychological tests (1.5 standard deviations below the normative mean) and the patient and/or care-partner voiced a subjective cognitive change but preserved independence with iADLs from a cognitive standpoint during the semi-structured clinical interview independently from their responses on the FAQ. The full test battery varied somewhat across patients and is described in detail elsewhere (Statucka and Cohn, 2019). The semi-structured interview is described in Statucka et al. (2023).

##### 2.3.2.3 Cognitive domains

Composite scores for each of five cognitive domains were derived for analyses. A subset of the tasks administered during the comprehensive neuropsychological assessment were included in our analyses if available for at least 90% of the sample, as the test battery used with individual patients varied to some extent. The executive function composite score included performance on the Matrix Reasoning subtest from the Wechsler Abbreviated Scale of Intelligence 2nd edition (WASI-II) (Wechsler, 2011), the Conditional Associative Learning Test (CALT) (Taylor et al., 1990), the Category Switching subtest from the Delis Kaplan Executive Function System (DKEFS) (Delis et al., 2001), the Wisconsin Card Sorting Test (WCST) (Heaton et al., 1993), and performance on either Color Trail Making Test 2 (D’Elia et al., 1996; *n* = 44) or Trail Making Test B (TMT-B) (Reitan, 1992; *n* = 243). The attention composite included scores on the Digit Span subtest of the Wechsler Adult Intelligence Scale 3rd edition (WAIS-III) (Wechsler, 1997), and Color Trail Making 1 (D’Elia et al., 1996; *n* = 44) or Trail Making Test A (TMT-A) (Reitan, 1992; *n* = 243). The memory composite included the Total Recall (immediate recall of trials 1–5), Long Delay Free Recall, and Recognition Discriminability measures on the California Verbal Learning Test 2nd edition (CVLT-II) (Delis et al., 2000), and the Rey-Osterrieth Complex Figure (ROCF) recognition trial (Meyers and Meyers, 1995). The language composite included scores on the Boston Naming Test (BNT) (Kaplan et al., 1983) and the Category Fluency subtest from the DKEFS (Delis et al., 2001). Lastly, the visuospatial skills composite included performance on the ROCF copy (Meyers and Meyers, 1995) and the Benton Judgment of Line Orientation test (JLO) (Calamia et al., 2011).

Raw test scores on these neuropsychological tests were converted to z-scores using age or age-and-education corrected normative data. We then windsorized extreme scores by replacing values < –3.0 z by scores of –3.0 z. This included changes on the CALT (*n* = 29), BNT (*n* = 27), Color Trails 1 or TMT-A (*n* = 13), Color Trails 2 or TMT-B (*n* = 11), and JLO (*n* = 2). Following this, missing values were replaced by the group’s median z-scores. This pertained to Matrix Reasoning (*n* = 1), CALT (*n* = 2), ROCF recognition (*n* = 18), BNT (*n* = 2), ROCF copy (*n* = 2), and JLO (*n* = 2). There were no missing values on the Trail Making tasks, and there were no extreme or missing values for Digit Span, CVLT-II, WCST, or the Category Fluency and Category Switching subtests of the DKEFS. Z-scores were then averaged over each of the five cognitive domains (attention, executive function, memory, visuospatial skills, and language) to create separate composite scores for each domain.

### 2.4 Statistical analysis

Analyses were run using R version 4.3.2 for MacOS (R Core Team, 2021). The *p-*value threshold was set to *p* = 0.05 for all analyses, with Bonferroni correction for multiple comparisons when applicable. Normality assumptions were tested using the Shapiro–Wilk test. Demographic variables were analyzed using the Mann Whitney *U-*test, except sex and depression severity, which were examined using the Pearson chi-square test. Average ratings for each symptom-type on the extended FAQ (i.e., degree to which iADLs are limited by motor symptoms and cognitive symptoms, respectively) were compared using Wilcoxon Signed Rank Tests (*W)* at three levels: (i) with all endorsed FAQ items grouped together, (ii) with endorsed FAQ items grouped into the categories proposed by Becker et al. (2020), and (iii) for each endorsed FAQ item individually. Effect sizes (Rosenthal’s *r* and Cramér’s *V*) are also reported. Comparisons between effect sizes were done using the Fischer r-to-z transformation.

We then examined each standard FAQ item’s relationship with measures of motor (UPDRS-III) and cognitive (DRS-2) functioning following a similar statistical approach as that used previously (Becker et al., 2020). Linear regressions were performed for each FAQ item against the UPDRS-III and DRS-2, with age, sex, and disease duration as covariates.

To investigate how the two measures of cognitive iADL impairment reflect current cognitive status, FAQ_Q_ and RCB scores were compared between diagnostic groups (PD-cognitively normal (PD-CN) vs. PD-MCI) using Mann Whitney *U-*tests. Lastly, we examined the relationship between iADL impairment and cognition in more detail using Spearman’s correlations of the FAQ_Q_ and RCB with each of the five cognitive domain composite scores derived from the neuropsychological assessment.

## 3 Results

### 3.1 Demographic and clinical characteristics

As shown in Table 1, of the 283 patients included, 119 (42%) were deemed to be cognitively normal (PD-CN) and 164 (58%) were diagnosed with PD-MCI. Patients with PD-MCI showed more severe motor impairment as assessed by the UPDRS-III and higher total scores on the standard FAQ rated by patients and care-partners. The PD-MCI group had more people with moderate-severe scores on a depression scale, and PD-CN group had more people who endorsed no depression symptoms. There were no significant differences in age, sex, education, disease duration, LEDD, or frequency of individuals with mild depression symptom severity between the diagnostic groups.

**TABLE 1:** Patient demographics and clinical characteristics.

Measure	Total sample (*n* = 283)	PD-CN (*n* = 119)	PD-MCI (*n* = 164)	PD-CN vs. PD-MCI statistics
Age (years)	61.83 (8.01)	61.76 (8.41)	61.89 (7.74)	*U* = 9,943, *p* = 0.79, *r* = 0.016
Male sex	63.60%	63.03%	64.02%	χ^2^ = 0.03, *p* = 0.86, *V* = 0.01
Education (years)	14.19 (2.86)	14.39 (2.90)	14.06 (2.83)	*U* = 10,274, *p* = 0.44, *r* = 0.046
PD duration (years)	10.35 (4.70)	10.11 (4.56)	10.53 (4.81)	*U* = 9,219, *p* = 0.43 *r* = 0.047
LEDD (mg)	1363.87 (584.91)	1346.25 (602.44)	1376.65 (573.37)	*U* = 9,312, *p* = 0.51 *r* = 0.039
UPDRS-III (ON)	14.64 (8.90)	12.74 (8.66)	16.02 (8.83)	***U* = 7,537, *p* = 0.001, *r* = 0.194**
FAQ self (0–30)	5.13 (5.01)	3.45 (3.71)	6.35 (5.46)	***U* = 6,420, *p <* 0.001 *r* = 0.294**
FAQ care-partner (0–30)	4.71 (4.63)	3.54 (3.34)	5.56 (5.23)	***U* = 7,720, *p* = 0.003 *r* = 0.179**
**Depression scale severity^a^**				
None	65.37%	78.15%	56.10%	**χ^2^ = 14.82, *p* < 0.001, *V* = 0.23**
Mild	21.55%	15.97%	25.61%	χ^2^ = 3.79, *p* = 0.05, *V* = 0.12
Moderate-severe	13.07%	5.88%	18.29%	**χ^2^ = 9.35, *p* = 0.002, *V* = 0.18**

^a^ Depression symptom severity was determined using scores on the Beck Depression Inventory (BDI-II; *n* = 267) or the Geriatric Depression Scale (GDS; *n* = 16). Results are reported as “mean (standard deviation)” or % of sample, PD, Parkinson’s disease; CN, cognitively normal; MCI, mild cognitive impairment; LEDD, Levodopa-equivalent daily dose; UPDRS-III (ON), Unified Parkinson’s Disease Rating Scale Part 3 in the ON medication state; FAQ, Functional Activities Questionnaire. Significance threshold was *p* < 0.05 corrected for multiple comparisons. Bold values indicate statistically significant results.

### 3.2 Motor and cognitive contributions to iADL difficulties

#### 3.2.1 Overall motor and cognitive contributions

Overall, motor symptoms were rated as more limiting to iADL function than cognitive symptoms by both patients [Motor: M = 1.56, SD = 0.93; Cognitive: M = 0.98, SD = 0.89; *W*(947) = 32,280, *p* < 0.001, effect size *r* = 0.454] and care-partners [Motor: M = 1.54, SD = 0.93; Cognitive: M = 0.81, SD = 0.88; *W*(902) = 38,058, *p* < 0.001, effect size *r* = 0.504].

#### 3.2.2 FAQ items by category

**FIGURE 2:**
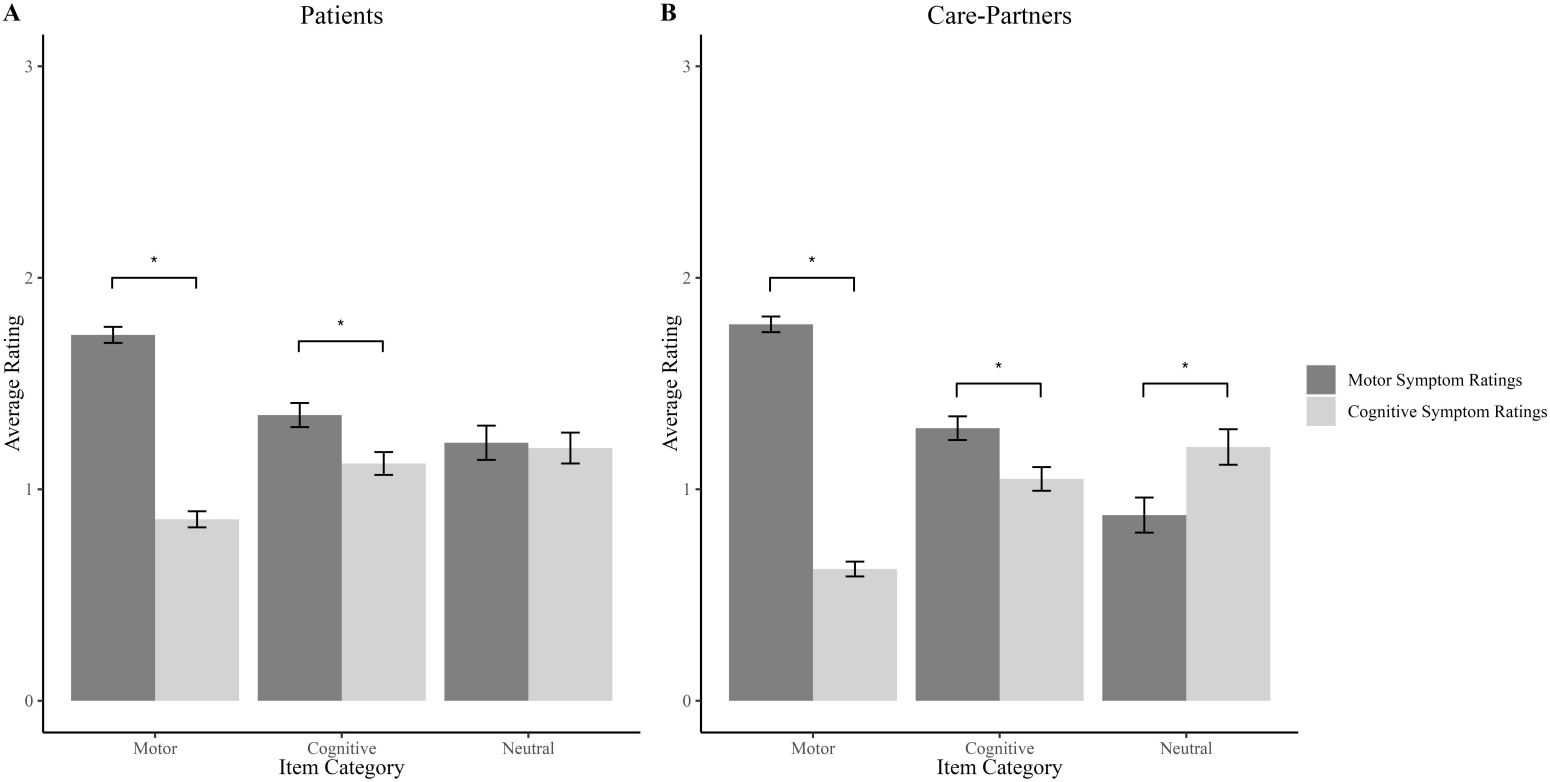
Attributed source of iADL difficulties for each modified scoring method item category. **(A)** Patient ratings **(B)** Care-partner ratings. * Indicates a statistically significant difference.

As shown in Figure 2, items categorized as motor in previous research (Becker et al., 2020) were reported to be more limited by motor symptoms than cognitive symptoms by both patients [*W*(562) = 5,218, *p* < 0.001, effect size *r* = 0.640] and care-partners [*W*(546) = 3,580, *p* < 0.001, effect size *r* = 0.742]. However, items previously categorized as cognitive were also reported to be more limited by motor symptoms than cognitive symptoms by both patients [*W*(262) = 4,492, *p* = 0.005, effect size *r* = 0.197] and care-partners [*W*(266) = 7,611, *p* = 0.008, effect size *r* = 0.169). Notably, these differences in limitations attributed to motor versus cognitive difficulties showed greater effect sizes for the motor items than for the cognitive items for both patients (*z* = 7.44, *p* < 0.001) and care-partners (*z* = 10.44, *p* < 0.001), which partly supports previous findings regarding their item classification to the motor category. For the neutral items, there was no significant difference between patients’ ratings for motor and cognitive symptoms [*W*(123) = 1,042, *p* = 0.83, effect size *r* = 0.014). However, care-partners rated neutral items as being significantly more limited by cognitive symptoms [*W*(90) = 1,128, *p* < 0.001, effect size *r* = 0.339).

#### 3.2.3 Cognitive and motor contributions per FAQ item

As reported in Table 2, all individual items previously categorized as motor (Becker et al., 2020) were reported to be significantly more limited by motor symptoms by both patients and care-partners. Results for the items categorized as cognitive (items 1, 2, and 9) were mixed, however. Contrary to previous research, item 1 (Cheques and bills) was rated as being more limited by motor symptoms by both patients and care-partners, while there were no significant differences in symptom type attribution for item 2 (Complex finances). Only item 9 (Remembering important events) aligned with previous findings as it was rated as being more limited by cognitive than motor symptoms, although this effect was only statistically significant for the care-partner ratings. Difficulties with neutral items 7 (Tracking current events) and 8 (Attention to media) were attributed to both motor and cognitive symptoms, with no significant difference between the ratings for each symptom type.

**TABLE 2:** Itemwise comparisons between the average motor and cognitive symptom ratings on the extended FAQ.

FAQ items	Patient ratings				Care-partner ratings			
	n	Motor symptom rating	Cognitive symptom rating	*p*	n	Motor symptom rating	Cognitive symptom rating	*p*
**Motor items**								
3 Shopping/errands	140	1.86 (0.92)	0.79 (0.87)	** <0.001**	133	1.96 (0.77)	0.50 (0.75)	** < 0.001**
4 Hobbies/skills	98	1.56 (0.85)	0.85 (0.95)	** < 0.001**	94	1.54 (0.88)	0.71 (0.79)	** < 0.001**
5 Making coffee	54	1.50 (0.89)	0.69 (0.59)	** < 0.001**	61	1.46 (0.94)	0.31 (0.62)	** < 0.001**
6 Preparing meals	118	1.60 (0.82)	0.87 (0.86)	** < 0.001**	140	1.71 (0.83)	0.57 (0.82)	** < 0.001**
10 Travel out of house	152	1.90 (0.95)	1.01 (0.94)	** < 0.001**	153	1.95 (0.87)	0.83 (0.92)	** < 0.001**
**Cognitive items**								
1 Cheques and bills	82	1.60 (0.86)	0.90 (0.92)	** < 0.001**	98	1.63 (0.83)	0.68 (0.87)	** < 0.001**
2 Complex finances	92	1.48 (0.93)	1.25 (0.90)	0.08	86	1.41 (0.86)	1.16 (0.96)	0.06
9 Remember imp. event	88	0.99 (0.86)	1.19 (0.79)	0.04	82	0.76 (0.81)	1.37 (0.78)	** < 0.001**
**Neutral items**								
7 Tracking current events	68	1.18 (0.93)	1.18 (0.59)	0.10	45	0.84 (0.80)	1.16 (0.88)	0.04
8 Attention to media	55	1.27 (0.87)	1.22 (0.83)	0.07	45	0.91 (0.79)	1.24 (0.71)	0.009

FAQ, Functional Activities Questionnaire. Results are reported as “mean (standard deviation).” Significance threshold was *p* < 0.05 corrected for multiple comparisons. Bold values indicate statistically significant results.

### 3.3 Relation to UPDRS-III and DRS-2 scores

As reported in Table 3 patients’ ratings on items previously categorized as motor were positively correlated with the UPDRS-III score, apart from item 4 (Hobbies/skills), for which the correlation did not survive the correction for multiple comparisons. Care-partners’ ratings on two of the five motor items showed a significant correlation with the UPDRS-III [items 3 (Shopping/errands) and 10 (Travel out of the house)]. Notably, none of the cognitive or neutral items were significantly associated with the UPDRS-III.

**TABLE 3:** Relationship between FAQ items and independent measures of motor and cognitive functions.

FAQ item	UPDRS-III				DRS-2			
	Self		Care-partner		Self		Care-partner	
	*r*	*p*	*r*	*p*	*r*	*p*	*r*	*p*
**Motor items**								
3 Shopping/errands	0.281	**< 0.001**	0.337	**< 0.001**	–0.255	**0.001**	–0.295	**< 0.001**
4 Hobbies/skills	0.202	0.02	0.141	0.23	–0.127	0.34	–0.096	0.63
5 Making coffee	0.280	**<0.001**	0.218	0.01	–0.255	**0.001**	–0.179	0.06
6 Preparing meals	0.267	**<0.001**	0.197	0.03	–0.280	**<0.001**	–0.170	0.09
10 Travel out of house	0.329	**<0.001**	0.247	**0.001**	–0.355	**<0.001**	–0.301	**<0.001**
**Cognitive items**								
1 Cheques and bills	0.164	0.11	0.225	0.01	–0.194	0.03	–0.257	**0.001**
2 Complex finances	0.164	0.11	0.190	0.04	–0.134	0.28	–0.244	**0.002**
9 Remember imp. event	0.178	0.06	0.167	0.09	–0.207	0.02	–0.176	0.07
**Neutral items**								
7 Tracking current events	0.214	0.01	0.116	0.44	–275	**<0.001**	–0.181	0.05
8 Attention to media	0.191	0.03	0.080	0.78	–0.175	0.07	–0.162	0.12

FAQ, Functional Activities Questionnaire; UPDRS-III (ON), Unified Parkinson’s Disease Rating Scale Part 3 in the ON medication state; DRS-2, Dementia Rating Scale-2; r, correlation coefficient. Significance threshold was *p* < 0.05 corrected for multiple comparisons. Bold values indicate statistically significant results.

In keeping with previous cognitive classification, DRS-2 scores were negatively correlated with ratings for items 1 (Cheques and bills) and 2 (Complex finances) but only when rated by care-partners when corrected for multiple comparisons. However, this effect was not seen for cognitive item 9 (Remembering important events). Moreover, DRS-2 scores were also associated with patients’ and care-partners’ ratings for a number of motor items [Patient ratings for items 3 (Shopping/errands, 5 (Making coffee), 6 (Preparing meals), 10 (Travel out of house); Care-partner ratings for item 3 (Shopping/errands) and item 10 (Travel out of house)].

Results were similarly mixed for the neutral items. In accordance with prior research (Becker et al., 2020), we found that neither patients’ nor care-partners’ ratings for item 8 (Attention to media) were correlated with either the UPDRS-III or DRS-2. However, while item 7 (Tracking current events) was previously associated with both UPDRS-III and MoCA scores, we found that only care-partners’ ratings for this item reached the statistical threshold for the relationship with the DRS-2 in our sample.

### 3.4 Validation of measures of cognitive iADL impairment

As reported in Table 4, the FAQ_Q_ did not differ between PD-NC and PD-MCI groups regardless of whether calculated using patient or care-partner ratings. However, patients diagnosed with PD-MCI had a higher RCB than those with intact cognition. These differences were noted both when calculated with patient ratings (*U* = 7,272, *p* < 0.001, effect size *r* = 0.223) and with care-partner ratings (*U* = 7,395, *p* < 0.001, effect size *r* = 0.218) on the extended FAQ. These patterns remain when we exclude people with moderate-severe depression scores (see Supplementary Table 1).

**TABLE 4:** Comparing measures of cognitive iADL impairment by cognitive status.

Measure		Median (IQR)		*p*	Effect size (*r*)
		PD-CN (*n* = 119)	PD-MCI (*n* = 168)		
FAQ_Q_	Patient	0.83 (0.40)	0.75 (0.43)	0.26	0.068
	Care-partner	0.75 (0.44)	0.82 (0.40)	0.11	0.096
RCB	Patient	0.11 (0.50)	0.40 (0.50)	**0.001**	**0.223**
	Care-partner	0.00 (0.33)	0.29 (0.46)	**0.001**	**0.218**

IQR, Interquartile Range; CN, Cognitively Normal; MCI, Mild Cognitive Impairment; FAQ_Q_, Functional Activities Questionnaire quotient; RCB, Reported Cognitive Burden. Significance threshold was *p* < 0.05 corrected for multiple comparisons. Bold values indicate statistically significant results.

As reported in Table 5, there were no statistically significant relationships between the FAQ_Q_ and average z-scores for each cognitive domain regardless of whether calculated using patient or care-partner ratings. In contrast, there were significant negative correlations between the RCB and average scores on tests of executive functioning and attention, both when calculated with patient and care-partner ratings on the extended FAQ.

**TABLE 5:** Relationships between cognitive iADL impairment and neuropsychological composite measures.

Cognitive iADL measure	Executive	Attention	Memory	Language	Visuo-spatial
**FAQ_Q_**					
Patient	0.030, *p* = 0.62	0.025, *p* = 0.68	–0.015, *p* = 0.81	0.066, *p* = 0.29	0.063, *p* = 0.29
Care-partner	–0.031, *p* = 0.61	–0.022, *p* = 0.72	–0.063, *p* = 0.29	0.084, *p* = 0.16	0.076, *p* = 0.21
**RCB**					
Patient	**–0.170, *p* = 0.004**	**–0.171, *p* = 0.004**	–0.121, *p* = 0.04	–0.043, *p* = 0.47	–0.143, *p* = 0.02
Care-partner	**–0.167, *p* = 0.005**	**–0.166, *p* = 0.005**	–0.145, *p* = 0.02	–0.117, *p* = 0.05	–0.088, *p* = 0.14

Reported results are Spearman’s correlation coefficients (*r*_*s*_). FAQ_Q_, Functional Activities Questionnaire quotient; RCB, Reported Cognitive Burden. Significance threshold was *p* < 0.05 corrected for multiple comparisons. Bold values indicate statistically significant results.

## 4 Discussion

We examined the validity of two approaches aimed at isolating the contribution of cognitive symptoms to iADL difficulties from that of motor symptoms based on responses on the FAQ (standard and extended versions) in PD patients with disabling motor symptoms and fluctuations. This distinction is a major challenge for clinicians and is critical for accurate cognitive diagnosis, prognosis, and intervention planning. We first applied a previously validated scoring method of the standard FAQ (Becker et al., 2020), which yields a quotient (FAQ_Q_) reflecting the ratio of difficulties on FAQ items associated with objective cognitive measures to those on FAQ items related to motor scores. We then used a novel extension of the FAQ that explicitly asks patients and care-partners to rate the extent to which each endorsed iADL difficulty is due to motor and cognitive causes, respectively, and then converted these ratings into the Reported Cognitive Burden (RCB) score. In our pre-DBS cohort, motor symptoms were most often identified as the greater source of limitation by both patients and care-partners, even for items previously classified as “cognitive” (Becker et al., 2020). The FAQ_Q_ did not differ significantly between patients with PD-MCI and those with intact cognition (PD-CN) in our sample, and its correlations with objective cognitive measures were weak and did not reach statistical threshold. Thus, our results did not replicate previous findings in a sample with more severe motor disability. In contrast, our new RCB score, derived directly from attributions based on lived-experience, was higher in PD-MCI than PD-CN, and related to performance in attention and executive domains, regardless of whether ratings were those of patients or of care-partners. Thus, the RCB offers a sensitive means of detecting mild functional changes related to cognition in the context of disabling motor symptoms, including changes that precede the overt loss of independence seen in PD dementia (PDD). As such, it may provide support in the diagnosis of cognitive dysfunction in PD. In the sections that follow, we discuss how these findings align with and extend prior literature on questionnaire-based iADL measurement in PD, alternative approaches to iADL assessment, strengths and limitations, and future directions.

Determining the impact of cognitive decline on one’s ability to function independently is essential when making a dementia diagnosis but there is increasing recognition that subtle changes in the ability to carry out cognitively demanding iADLs may be present even at earlier stages of PD (Kulisevsky et al., 2013). Therefore, there is a pressing need to assess functional impairment on a continuum, to capture even minor changes that may occur early in the disease course, and to sample various relevant activities rather than relying on a patient’s ability to perform any single activity (Kulisevsky et al., 2025). Using standardized questionnaires allows researchers and clinicians to address these needs in a time- and resource-efficient way. However, it is important to recognize that most existing questionnaires were designed for different patient populations (typically Alzheimer’s disease). As such, they either do not ask about the source of disability (physical, cognitive, emotional, behavioral) or focus on iADLs impacted by memory and disorientation but not necessarily those impacted by executive dysfunction which are most common in PD (Marras et al., 2014). As a result, there has been increased effort toward adapting existing questionnaires to better suit PD populations (Almeida et al., 2017; Cheon et al., 2015) or developing PD-specific questionnaires such as the Penn Daily Activities Questionnaire (PDAQ) (Brennan et al., 2016) and the Parkinson’s Disease Cognitive Functional Rating Scale (PD-CFRS) (Kulisevsky et al., 2013).

An alternative approach is to modify only the scoring of an established questionnaire as done by Becker et al. (2020). This has the advantage of preserving the psychometric properties of the original standardized questionnaire as there is no modification of the items or of the test administration, and this is less resource-consuming than developing and validating a new PD-specific measure. An additional strength is the potential prognostic value of this method. Indeed, in a longitudinal study (Becker et al., 2022a), the presence of cognitive iADL impairment (FAQ_Q_ > 1) combined with PD-MCI at baseline predicted conversion to PDD more strongly than PD-MCI alone (HR = 12.01 vs. HR = 5.34). Although innovative, the FAQ scoring method treats individual iADLs as binary (either cognitive or motor), but our results indicate that it is common for iADL performance to be impacted by more than one source of difficulty, as patients and care-partners rated both motor and cognitive symptoms as contributing to all iADLs on the extended FAQ. This is further supported by Benge and Balsis (2016), who showed that care-partners often identified multiple sources of difficulty when asked about the impact of physical, cognitive, and emotional/behavioral problems on iADLs. Thus, approaches that classify items exclusively as “motor” or “cognitive” may be too coarse.

Moreover, because our sample was derived from patients undergoing evaluation for DBS candidacy—a treatment indicated for individuals with motor fluctuations, pronounced OFF periods, levodopa-induced dyskinesias, or tremor inadequately controlled with pharmacotherapy—it is anticipated that motor disability would strongly contribute to iADL difficulties. Accordingly, the proposed FAQ_Q_ > 1 cutoff may be less applicable in this cohort, as cognitive symptoms are unlikely to overshadow the motor contribution to iADLs in this population. This is further illustrated by the fact that 15.5% of our sample had a FAQ_Q_ > 1 based on care-partners’ ratings versus 23.9% of the sample in Becker et al. (2022a) despite having higher rates of PD-MCI (58% vs. 29%), longer disease duration (10.4 vs. 4.1 years) and higher intake of dopaminergic medications (1,365 mg vs. 511 mg per day). Our RCB may provide a useful alternative, or addition to the FAQ_Q_, for addressing these challenges as it is derived from subjective ratings and represents cognitive contribution as a proportion of the overall difficulty rather than as a relative ratio between symptom types. Importantly, only the RCB and not the FAQ_Q_ differentiated PD-MCI from PD-NC in our pre-DBS population and was related to performance on tasks assessing executive functioning and attention, two domains that are particularly vulnerable in PD (Aarsland et al., 2021). These results contrast with prior ones using the FAQ_Q_ in non-surgical PD patients (Becker et al., 2020).

Although questionnaires are efficient and practical tools that can be readily utilized in routine clinical care, they inherently rely on self-report which may be influenced by respondents’ mood or level of insight, as well as by caregiver burden (Carlisle et al., 2023), potentially leading to either overestimation or underestimation of a patient’s functional abilities (Shulman et al., 2006; Pirogovsky et al., 2014). To provide a more objective assessment of how cognition impacts daily functioning, the Movement Disorder Task Force previously recommended using the Pill Questionnaire which asks patients to verbally describe their medication schedule (Dubois et al., 2007). If the patient can successfully identify the medications and their timing, cognition is considered to have no impact on daily living. However, patients’ declarative memory of their medication regimen may not necessarily match their daily medication adherence. Moreover, medication regimens can differ greatly across patients in terms of complexity and the frequency with which changes are made by their physician, making the difficulty of this task inconsistent. Also, while medication management is a particularly salient and repetitive part of disease management, this questionnaire might not reflect changes in other functional domains. Given these caveats, it is perhaps not surprising that this measure failed to diagnose around half of the probable PDD patients compared to neurologists’ assessments in one study (Lee et al., 2014). Notably, the patients diagnosed only by neurologists had lower motor symptoms and higher cognitive functioning, suggesting the Pill Questionnaires may lack sensitivity and only capture individuals on the more severe end of the PDD spectrum.

Performance-based tasks are standardized measures where patients are asked to perform one or more iADLs in a laboratory setting. They present an attractive alternative to questionnaires because of their standardized assessment and scoring, increased ecological validity, minimal reliance on patient or care-partner insight, and focus on real-life skills that may be targets for intervention (Pirogovsky et al., 2014). On these measures, PD patients perform more poorly on tasks with high cognitive demands such as “mock” medication (Shulman et al., 2006; Manning et al., 2012; Foster, 2014; Pirogovsky et al., 2014; Sumida et al., 2021; Schmitter-Edgecombe et al., 2022) and financial management (Martin et al., 2013; Pirogovsky et al., 2014; Ariesen et al., 2024). Poor performance on these tasks is often related to a variety of cognitive abilities (Foster, 2014; Holden et al., 2018; Fellows and Schmitter-Edgecombe, 2019; Sumida et al., 2021) including attention and executive functioning (Manning et al., 2012; Glonnegger et al., 2016; Sulzer et al., 2020; Schmitter-Edgecombe et al., 2022). However, correlations with cognitive abilities are not always replicated (Pirogovsky et al., 2014; Ariesen et al., 2024), and performance can also be related to the severity of motor symptoms (Manning et al., 2012; Foster, 2014; Glonnegger et al., 2016; Holden et al., 2018; Fellows and Schmitter-Edgecombe, 2019) and advanced age (Manning et al., 2012; Foster, 2014). Therefore, although performance-based measures are enticing, they are also imperfect indicators of cognitive iADL functioning. It is important to consider that patients are asked to perform these assessments outside of their home environment where they have established routines, and may be able to use compensatory strategies, assistive devices, and environmental cues to improve their functioning (Shulman et al., 2006; Pirogovsky et al., 2014; Schmitter-Edgecombe et al., 2022). Tasks performed in a laboratory are likely less emotionally salient than those performed at home with real consequences for functioning and independence (e.g., sorting beans into a dosette versus taking one’s own medications as prescribed which improve motor symptoms). Performance-based measures may also underestimate everyday ability as individuals may perform more poorly when they are aware that they are being observed and evaluated. Finally, logistical challenges such as lack of dedicated space to set up a faux environment and the time-consuming nature of many of these tasks limit their utility in clinical settings.

Our study has several strengths such as including a sample of consecutive and diverse PD patients (see Statucka and Cohn, 2019) with advanced disease who underwent comprehensive neuropsychological assessments with questionnaires completed by both patients and care-partners. Our recent work (Statucka et al., 2023) with an overlapping sample also highlighted the importance of considering patients’ and care-partners’ lived-experience, which we have once again done with our extension of the FAQ. While PD is an extreme case of physical disability, our method could also be applied to other populations who have both physical and cognitive limitations such as cerebrovascular disease (Babulal et al., 2015; Blomgren et al., 2019) or elderly populations with sensory impairments (e.g., vision and hearing loss) or orthopedic issues (Liu et al., 2016; Shin and Kim, 2022; Zhou et al., 2025).

In terms of limitations, while the use of consecutively collected clinical data within a universal health care system may be less prone to recruitment biases than prospective research studies, it results in a sample that is not representative of a general PD population. As our patients are considering DBS surgery for treatment of their severe motor symptoms and fluctuations, the degree to which motor and cognitive symptoms contribute to their iADL difficulties likely differ from other subsets of PD patients. Due to the large impact of motor symptoms on daily living, pre-DBS patients might underestimate the effect of cognitive symptoms or have less opportunity to engage in certain activities, making it difficult to estimate the degree to which other sources contribute. It is also possible that patients and care-partners tailor their responses in a way that they hope may be favorable to their DBS candidacy determination (e.g., perhaps minimizing cognitive difficulties and/or amplifying the negative impact of motor symptoms to functioning). Moreover, in our center, patients with clear dementia are excluded early in DBS evaluation process, prior to the completion of our comprehensive neuropsychological assessments where we collect FAQ data. Thus, we are unable to evaluate the diagnostic precision of the RCB or FAQ_Q_ in terms of PDD.

Another limitation is that we did not account for other variables such as mood in our analyses, though there is evidence that depressive symptoms are a strong predictor of iADLs in both the general PD and pre-DBS patient populations (Choi et al., 2019; Bezdicek et al., 2022). Although our results regarding the relationship of the novel RCB with cognitive status remained even after excluding individuals reporting a moderate-severe level of depressive symptoms on questionnaires (Supplementary Table 1), such scales do not provide a formal diagnosis of depression. Additionally, depression questionnaires are not specific to mood as they include items also related to PD symptoms such as subjective cognitive complaints, sleep disturbances, and autonomic symptoms. Thus, future research should examine how formally diagnosed mood disorders relate to iADL difficulties in PD. Finally, this study was cross-sectional and thus, we could not determine the prognostic value of having a higher RCB or FAQ_Q_, nor investigate how pre-surgical motor and cognitive functions contribute to iADLs changes post-DBS. Thus, we aim to examine this approach in post-surgical patients who are likely to demonstrate improved motor symptoms and daily function, but perhaps cognitive decline following DBS. Future longitudinal studies should also aim at validating this method in non-surgical PD cohorts across cognitive diagnoses (i.e., intact cognition, PD-MCI, PDD) and disease stages, to examine to potential utility of this tool in the care of the broader PD population.

Our study once again emphasizes the importance of considering patients’ and care-partners’ lived-experiences to estimate the degree to which cognition impacts patients’ functional independence. The fact that similar results were obtained with patients’ and care-partners’ ratings is reassuring as it demonstrates that patients have sufficient insight into their own difficulties, and that their impressions should be considered especially when a knowledgeable care-partner is not available. Potential future extensions and applications of our findings are threefold. First, beyond patients with PD, our extended FAQ, and the RCB in particular, could be useful with other medically complex populations experiencing both physical and cognitive limitations (e.g., sensory or physical disability). Second, clarifying the impact of various symptoms on daily functioning is not only important for accurate cognitive diagnosis, but more broadly may inform treatment planning and engagement. For example, if a patient attributes their functional difficulties solely to physical symptoms, they may be unwilling to engage in cognitive remediation or treatment aimed at mood. Understanding patients’ attributions of their functional difficulties may lead to more personalized intervention plans with better outcomes for the individual. Finally, having a better understanding of the degree to which cognitive versus physical symptoms interfere with iADLs may also improve clinicians’ ability to identify individuals at elevated risk of developing dementia.

## Data Availability

The datasets presented in this article are not readily available because it consists of clinical data, and patients did not consent to data sharing. Requests to access the datasets should be directed to corresponding author, melanie.cohn@uhn.ca.
