# Supplementary material for: Separating cognitive and motor contributions to iADL difficulties in Parkinson’s disease
Source: Front Aging Neurosci. 2026 Jan 8;17:1732479. doi: 10.3389/fnagi.2025.1732479 (PMC12823802; doi:10.3389/fnagi.2025.1732479)
Supplement: Supplementary file 1 [file Table_1.docx]

***Supplementary Material***

***Supplementary Table S1:*** *Comparing measures of cognitive iADL impairment by cognitive status in subgroup with no or mild depression scores (BDI-II scores < 20)*

| **Measure** | | **Median (IQR)** | | **p** | **r** |
| --- | --- | --- | --- | --- | --- |
|  |  | **Intact Cognition (n=112)** | **PD-MCI**  **(n=134)** |  |  |
| **FAQ_Q_** | **Patient** | 0.83(0.40) | 0.75(0.43) | .449 | .048 |
|  | **Care-partner** | 0.75(0.50) | 0.85(0.40) | .047 | .127 |
| **RCB** | **Patient** | 0.00(0.42) | 0.36(0.50) | **<.001** | **.228** |
|  | **Care-partner** | 0.00(0.28) | 0.24(0.44) | **<.001** | **.220** |

*Note:* Patients reporting moderate-severe levels of depression (BDI-II ≥20) were excluded (n=37).

IQR = Interquartile Range, PD-MCI = Parkinson’s Disease Mild Cognitive Impairment, FAQ_Q_ = Functional Activities Questionnaire quotient, RCB = Reported Cognitive Burden, BDI-II: Beck Depression Inventory 2^nd^ edition
